# Solving the Insoluble: Regulation of α-Polyglucan Solubility and Functionality in Nature

**DOI:** 10.3390/molecules31193453

**Published:** 2026-09-28

**Authors:** Victoria Butler, Saheda Tamanna, Ian J. Tetlow

**Affiliations:** Department of Molecular and Cellular Biology, College of Biological Science, University of Guelph, Guelph, ON N1G2W1, Canada

**Keywords:** α-glucan, glucan phosphorylation, glycogen, branching enzymes, α-polyglucans, polymer solubility, starch

## Abstract

Carbohydrate storage in the form of α-polyglucans plays essential roles in fitness and survival of organisms from all kingdoms of life. Optimal biological functionality of carbohydrate storage reserves in different cellular environments requires strict control of specific physicochemical and biochemical parameters, such as water solubility and susceptibility to hydrolytic and phosphorolytic digestion. This review focuses on the major storage α-polyglucans found in nature: glycogen, plant and floridean starches, and semi-amylopectin, and how each are modified in different cells to maximize efficient storage and access carbon reserves. We highlight the important role of branching to induce and maintain water solubility or insolubility. The tendency of proximal long linear α-1,4-linked chains to interact and form water-insoluble starch granules in plants and green algae (Archaeplastida) is through controlled formation and removal of specific α-1,6-branch linkages. Conversely, the stability of water-soluble glycogen particles is compromised when there is an imbalance between α-glucan elongation and branching activities, leading to excess long chains prone to insolubility. In Archaeplastida starches, α-glucan phosphate esters are thought to disrupt neighboring chains to prime granules for amylolytic degradation, whereas their role in glycogen metabolism remains an open question. We discuss current knowledge of the functional significance of these modifications to α-polyglucan architecture.

## 1. Introduction

This article reviews our current perspective on structural modifications of glycogen and starch, two α-polyglucans that represent the major forms of osmotically inert carbohydrate storage found in the natural world. Both polymers are chemically identical, being made up of linear α-1,4-O-linked glucan chains and variable numbers of α-1,6-branch linkages, yet possess physiochemically distinct properties, which are tailored to their respective biological roles. The following section is a brief and general overview of the synthesis and structure of glycogen in different prokaryotic and eukaryotic organisms, and of starch from plants and green algae. For the purposes of this review, we define α-polyglucans as complex macromolecular α-glucan-based polymers. Also included is mention of related α-polyglucans, such as plant phytoglycogen, and floridean starches and semi-amylopectins of aquatic eukaryotes. Detailed expositions of glycogen and starch biosynthesis and metabolism are beyond the scope of this work, and we refer the reader to recent reviews on glycogen [1,2,3] and starch [4,5,6].

### 1.1. Glycogen Structure

Glycogen is a versatile storage α-polyglucan found in almost all organisms across five of the six kingdoms of life from archaea to mammals, as well as primitive autotrophs such as cyanobacteria and some Rhodophytes [1,2,3,7]. The smallest discrete glycogen structures are called β-particles and are typically 20–40 nm in diameter, with size variation dictated, to a degree, by the biological source, and in multicellular organisms by tissue or organ type. Aggregates of β-particles occur in many tissues and organisms and are known as α-particles, which have a lower surface area: volume ratio [3]. Physical constraints also limit size such that polymer growth beyond 25–30 nm diameter is probably limited by steric hinderance of glycogen biosynthetic machinery for the growing glycogen particle [8,9,10]. Individual particles contain as many as 55,000 glucose (Glc) units polymerized into linear α-1,4-linked chains with a relatively high frequency (~7–18%) of α-1,6-branch linkages [11]. Unlike starch, where branching is controlled and restricted, with branch points found as discrete clusters, branch points in glycogen appear more open, and apparently random, and this arrangement prevents the formation of the more ordered and tightly compacted structures found in amylopectin, such as α-glucan chains, which form a double helix [10]. Some studies point to the branch linkages in glycogen as being arranged in multiple (up to twelve) tiers [11], whereas structural studies of mouse (Mus musculus) liver glycogen argue for a random distribution of α-1,6-linkages [12,13]. Larger assemblies of β-particles, known as α-particles, are found in many organisms, from unicellular prokaryotes to complex eukaryotes [3,14,15]. Disruption of normal glycogen metabolism results in a number of well-known glycogen storage diseases, some of which are characterized by the formation of polyglucosan bodies (PGBs) in which the normal glycogen structure is altered, in some cases resembling plant starch [16,17].

### 1.2. Glycogen Metabolism

A general and simplified scheme of glycogen metabolism is shown in Figure 1 and is comprised of a relatively small body of enzymes committed to synthesis and degradation. However, control of carbon entry into, and release from, glycogen reserves operate through various complex post-transcriptional and post-translational mechanisms and signaling networks, which vary in different organisms and are beyond the scope of this review. The reader is referred to recent reviews on the regulation of glycogen metabolism in muscle tissue of eukaryotes [18], mammalian storage diseases [19], and glycogen metabolism in bacteria and fungi [2]. In eukaryotic cells glycogen biosynthesis occurs in the cytosol and is initiated through the action of a dimeric proteinaceous primer called glycogenin (GLG; EC 2.4.1.186), which covalently binds via autocatalysis Glc from UDP-Glc to a hydroxyl group on a conserved tyrosine residue [20,21,22]. The UDP-Glc precursor is synthesized from UTP and glucose 1-phosphate (Glc1P) via UDP-Glc pyrophosphorylase (UGPase; EC 2.7.7.9). However, in archaebacteria and most bacteria, the nucleotide diphosphate sugar donor for glycogen synthesis is ADP-Glc, synthesized from ATP and Glc1P by ADP-Glc pyrophosphorylase (AGPase; EC 2.7.7.27), a highly regulated enzyme allosterically activated by fructose 1,6-bisphosphate and inhibited by AMP [23,24]. Once a chain of up to seven glucosyl residues are attached to GLG via the reducing end, the polymer is primed for glycogen synthesis. Prokaryotic cells do not possess GLG, and it is thought that the initiation of glycogen synthesis occurs via a self-priming mechanism associated with glycogen synthase (GS; EC 2.4.1.11) [25]. Eukaryotic GS (and some bacterial GSs, termed GlgA) elongates α-glucan chains by transferring glucosyl units from UDP-Glc to the non-reducing end of extant α-1,4-O-linked chains. Bacterial and archaebacterial GSs utilize ADP-Glc for chain elongation. Glycogen branching enzymes (GBEs; EC 2.4.1.18) play a crucial role in the formation of the water-soluble glycogen particle by shortening long α-glucan chains produced by GSs, and re-introducing these chains with α-1,6-branch linkages. The action of GBE has a profound effect on the physicochemical properties of the α-polyglucan and its ability to be degraded [26,27,28]. The glycogen particle grows through the concerted actions of GS and GBE, and the ratio of the two activities appears to be important for the formation of a normal particle structure. The density of shorter glucan chains has been shown to increase toward the periphery of the nascent structure [9,29,30,31], making the polymer less accessible to further glucan chain elongation or branching. Studies with yeast GBE (Glc3) show that as polymer branching continues, the glucan becomes a less favorable substrate, perhaps leading to self-regulation of particle size, as observed in glycogen storing organisms [31]. GBEs from different biological sources show distinct rates of branching and varied chain lengths (degree of polymerization, DP) of branches produced [32]. The implications of these activities on the resulting α-polyglucan in terms of solubility and resilience to degradation are discussed below.

An important feature of glycogen’s flexibility as a carbon reserve is its rapid mobilization in response to metabolic energy demands. The linear α-1,4-chains of glycogen are degraded phosphorolytically by the enzyme glycogen phosphorylase (GP; EC 2.4.1.1), releasing Glc1P. α-1,6-branch linkages must be removed for complete phosphorolytic breakdown of the particle. In prokaryotes, debranching of α-1,6-branch points is catalyzed by isoamylase (GlgX, and in plants this is abbreviated to ISA; EC 3.2.1.68), by direct removal of the α-1,6-branch linkage, and is thus termed a direct debranching enzyme [33]. In eukaryotic cells removal of glycogen branch points is catalyzed by an indirect glycogen debranching enzyme (GDBE; EC 2.4.1.25/3.3.1.33) via a two-step process, which utilizes the products of GP activity (phosphorylase-limit dextrin, ΦLD). The short branch chains of ΦLD (~4 DP) are cleaved by GDBE, leaving a single α-1,6-linked glucosyl residue and a short free glucan chain (~3 DP), which is transglycosylated and attached to a neighboring linear α-1,4-linked chain for degradation by GP, and the remaining α-1,6-linked glucosyl unit is then debranched, releasing free Glc [34,35].

### 1.3. Starch Structure

The formation of water-insoluble starch granules inside plastids of plant and green algal cells plays a central role in the carbon economy and fitness of these organisms. Starch acts as both a short-term carbon store over a diurnal cycle in photosynthetic tissues, such as leaves (transient starch), as well as a longer-term storage in seeds and tubers for future generations (storage starch). In autotrophs, starch formation and turnover is an integral link between photosynthetic carbon fixation and nocturnal growth and development [5,36,37]. Starch granules are orders of magnitude larger (0.1–100 μm) than glycogen particles (20–40 nm) and are characterized by a complex, ordered internal architecture [38,39]. Starch is a composite of two polymers, amylose, a linear α-1,4-linked polymer representing a minor component [40], and the branched, major component, amylopectin, whose structural organization defines the physicochemical properties of the granule, with an average chain length of 17–18 DP [41]. Unlike the open, water-soluble glycogen particle, which is formed through the even distribution of a relatively high proportion of branches, the branch points in amylopectin are clustered in a non-random manner and represent approximately 5% of total glucosyl linkages [42,43,44,45]. Clustering of α-1,6-branches in amylopectin allows self-assembly of short- and intermediate-length α-glucan chains (10–12 DP) to form left-handed double helices excluding free water [46,47,48,49]. The clustered helical assemblies of amylopectin chains give rise to different higher-order structures (allomorphs or polymorphs) that can be distinguished using wide-angle X-ray diffraction [50,51,52]. Different allomorphs are related to biological functionality; for example, in long-term storage starches (e.g., from cereal endosperms) the α-glucan chain double helices are very tightly packed together (termed A-allomorph). Tissues and organs that regularly turn over starch reserves, e.g., transient leaf starch, tubers, rhizomes, and storage root starches, have more loosely packed, hydrated clusters of helical assemblies, and these starches are classed as B-allomorph (tubers and storage roots), whilst the C-allomorphs (leaves and cotyledons) are a physical mixture of A- and B-type crystalline allomorphs [53,54].

### 1.4. Starch Metabolism

The process of starch biosynthesis takes place inside specialized sub-cellular organelles termed plastids, ultimately derived from the process of endosymbiosis [55,56,57]. The complex process of starch granule initiation is distinct from that of glycogen and requires the coordinated actions of multiple enzymes and regulatory proteins within specific micro-environments in the plastid. The granule grows from a central zone termed the hilum, which is thought to share structural characteristics similar to glycogen [58]. The various enzymes and proteins involved in granule initiation influence granule morphology, size, and granule number [59]. Several recent reviews outline our current understanding of this process [60,61,62,63]. The soluble substrate for starch synthesis is ADP-Glc synthesized by AGPase, which in plants is subject to many levels of regulation [64,65]. Unlike glycogen synthesis, which utilizes a single isoform of GS and GBE for elongation and branching, respectively, amylopectin is formed through the concerted actions of multiple chain-elongating enzymes, termed starch synthases (SSs; EC 2.4.1.21) and starch branching enzymes (SBEs; EC 2.4.1.18; Figure 1). At least three SSs are involved in chain elongation, SSI, SSII, and SSIII, which each elongate gradually longer α-1,4 linear chains utilizing ADP-Glc. The amylose component of starch is synthesized by a granule-bound starch synthase (GBSS) within a pre-existing amylopectin matrix [40]. In most plants and green algae, branching occurs through the actions of two isoforms of SBE, SBEI, which forms longer branches (DP 10–16), and SBEII, responsible for short- to medium-length branched chains (DP 6–8) [32]. The multiple forms of SS and SBE are responsible, in part, for the polymodal chain-length distribution observed in plant and algal starches [43]. The SSs and SBEs also possess tissue-specific isoforms with distinct properties, and studies in many plants have shown that the various SSs and SBEs are regulated by protein phosphorylation and form various functional heteromeric protein complexes [66,67,68]. The precise role of these protein complexes is not clear, but disruption of such protein complexes in cereals has significant structural implications for amylopectin assembly [69]. Plant isoamylases (ISAs; EC 3.2.1.68) are a class of direct debranching enzyme (DBE) that play a critical role in starch synthesis by determining the semi-crystalline, water-insoluble properties of amylopectin [70,71,72,73,74]. Two isoforms, ISA1 (catalytically active) and ISA2 (a catalytically inactive regulatory isoform), are involved in amylopectin synthesis by trimming excess and inappropriately positioned α-1,6-branch linkages [72,75,76]. The hydrolysis and removal of selective branches facilitate local α-glucan chain interactions, α-helix formation, local exclusion of water, and formation of semi-crystalline structures as neighboring chains reach <10 DP [47,77]. A third ISA, ISA3, is involved in α-1,6-branch linkage removal during starch degradation and has retained its probable ancestral role in α-glucan turnover [78,79,80]. The important role played by ISA1/ISA2 in controlling branch point frequency and distribution for starch granule formation is exemplified by mutations of these enzymes, which lead to the formation of the water-soluble polymer phytoglycogen (see Section 1.4). ISA1 mutants from several species produce small quantities of starch, which can be abolished in mutants lacking all DBEs (ISA and limit dextrinase; EC 3.2.1.142) [81]. Further loss of α-amylase3 (AMY3; EC 3.2.1.1) in these DBE mutants restores starch synthesis and suggests ISA promotes the formation of semi-crystalline structures at the granule periphery, which are shielded from hydrolytic attack by amylases [75]. For more detailed expositions of starch biosynthesis in plants, the reader is referred to several recent reviews [4,6,82].

### 1.5. Phytoglycogen

Phytoglycogen is a water-soluble α-polyglucan made in *isa1*^−^ mutants of plants and green algae, which are essentially devoid of starch due to ISA1 being either absent or catalytically inactive. The mutation was first identified and characterized in sweetcorn as the *sugary-1* gene knockout [72,83]. Other, less common sources of phytoglycogen are found in heterotrophic plastids of Müllerian bodies from myrmecophyte tropical trees, such as *Ryparosa kurrangii* [84] and *Crecropia peltata,* and result from the tissue-specific downregulation of ISA1 genes [85]. Structurally, phytoglycogen closely resembles normal glycogen, being present as discrete particles of 30–100 nm diameter with α-glucan chains branched in a dendritic manner at a frequency of 8–9% [41,86,87], resulting in a polyglucan with unusually high molecular density and potentially useful physicochemical properties [30,88]. The average glucan chain length of phytoglycogen is approximately 11–12 DP, with a denser distribution of chains at the polymer surface [29,30]. The relatively uniform physical structure of phytoglycogen from many biological sources and its similarity to glycogen structure is suggestive of physicochemical self-regulation of the particle size similar to that noted for glycogen described above. Analysis of these structures underlines the importance of the control of branching frequency and branch point positioning in starch granule formation by isoamylases. In rice endosperm, mutant forms of ISA1 showing reduced catalytic activity and disrupted interactions with the non-catalytic, regulatory, ISA2 isoform produce phytoglycogen with altered structural characteristics, e.g., increased particle diameter [89], further emphasizing the importance of the regulation of branch point positioning in determining the overall polymer structure.

### 1.6. Floridean Starch and Semi-Amylopectin

A variety of organisms synthesize water-insoluble starch-like storage α-polyglucans with α-1,4- and α-1,6-linkages, which are termed floridean starch and semi-amylopectin. Floridean starch is synthesized by members of the Florideophyceae class of red algae (Rhodophyta) and glaucophytes [90] and is similar in structure to the semi-amylopectins produced by other Rhodophyte groups and other organisms, such as some cyanobacteria [91]. Semi-amylopectin is characterized by having structural features intermediate between plant starch and glycogen [90]. Specifically, water-insoluble semi-amylopectins are composed of long chains (DP ≥ 20) and short chains (DP ≤ 8) in proportions intermediate between water-insoluble starch and water-soluble glycogen (see Figure 2). Unlike normal plant starches in which amylose is found as a variable, minor component of the granule, in Rhodophyte floridean starch and cyanobacterial semi-amylopectins, the presence of amylose is not universal. Floridean starches are considered to be amylose-free, based on analyses of Florideophyceae, such as *Gracilariopsis* sp. [92], although in other groups of Rhodophytes, such as the Porphyridiales, e.g., *Porphyridium purpurem*, the semi-amylopectin of some of the organisms in this order also contains amylose [90]. There is wide variation in the types of storage α-polyglucans within the Porphyridiales, for example, primitive Rhodophytes within this group, e.g., *Cyanidoschyzon merolae*, produce amylose-free semi-amylopectin, and others, such as *Galdieria sulphuraria* and *Cyanidium caldarium*, produce glycogen [93]. This is particularly interesting, as *Cyanidioschyzon merolae, C. caldarium*, and *G. sulphuraria* all inhabit similar extreme environments (i.e., hot acidic springs). Cyanobacteria show analogous variation in their modes of polyglucan storage, some species producing semi-amylopectin (*Myxosarcina burmensis* and *Synochococcus* sp.) and amylose-free semi-amylopectin (e.g., *Cyanobacterium* sp. MBIC10216), whilst others store water-soluble glycogen [91]. The fine structures of floridean starches, in terms of chain-length distribution of the amylopectin, are similar to plant and green algal starches, indicative of close structural relationships between these polyglucans [92,94]. X-ray diffraction patterns of granules from these organisms suggest lower levels of crystallinity and order in floridean starch compared with plant starches [90,92,94,95,96].

The general pathway for water-insoluble α-glucan formation in these cells is similar to plants and algae in that α-1,6-linkage formation is controlled through debranching enzymes. However, there are a number of key differences, which are of interest in terms of understanding the evolution of the starch biosynthesis pathway. Rhodophytes are amongst the most primitive eukaryotes [97], and the floridean starch pathway reflects this in that granules are synthesized in the cytosol (same as glycogen metabolism in animals and fungi), in some cases via a UDP-Glc-dependent pathway [98].

## 2. Physical and Biological Effects of α-Glucan Branching

The introduction of -1,6-branch linkages into polyglucans by SBEs and GBEs has profound effects on the solubility and biological functionality of the resulting polymer. Specifically, branch point distribution and branching frequency are important factors affecting water solubility and resilience to hydrolytic and phosphorolytic attack (digestibility). To some extent these factors are properties of individual branching enzymes from the various kingdoms, which are beyond the scope of the current review. Rather, the following section deals with the physical and biological consequences of α-glucan branching in plants and other organisms. For information on the key properties of branching enzymes, the reader is referred to several pertinent reviews and research articles [32,99,100,101,102].

### 2.1. Branching of Amylopectin in Starch Granules

Water-insoluble amylopectin is the structure-defining component of plant starch, semi-amylopectin, and floridean starch, representing approximately 75% of the granule mass in many plant species, with the remainder of the polymer consisting of unbranched amylose. The water-insoluble nature of amylopectin is a product of the tight control of both branching frequency and the resulting branch chain lengths, chiefly brought about through the actions of SBEs, which introduce branch points of varying chain length, and debranching enzymes (ISA1 and ISA2), which limit branch frequency in a non-random manner. For a water-insoluble, crystalline-competent structure to form, a number of tightly restricted physicochemical parameters appear to be required. All amylopectins have a low branching frequency in comparison to soluble polyglucans, such as glycogen and phytoglycogen. For most normal amylopectins, this branching frequency ranges between approximately 4.6% and 5.4% [42,103,104,105]. Amylopectin with an extremely reduced branching frequency has been produced in plants where the expression of dominant SBE isoforms (SBEII class in cereals and grasses) has been suppressed, e.g., the *amylose extender* (*ae*^−^) mutants in cereals, where branching frequency can become as low as 2.14% [69,104]. Starches with a low branching frequency show resistance to amylolytic (AMY) digestion in the gut and contribute to dietary-resistant starch, which is associated with a number of health benefits [106,107,108]. Low branching frequency in starch correlates with higher average chain lengths of amylopectin as well as elevated α-glucan phosphate levels [82] (see Section 3.1). In addition to branching frequency, the glucan chain-length distribution of various polyglucans can determine solubility (see Figure 2). Analysis of α-polyglucans in relation to their proportions of relatively long glucan chains (DP ≥ 20) and short glucan chains (DP ≤ 8) reveals distinct structural parameters required for water solubility or water insolubility (Figure 2). All water-insoluble starches and starch-like polymers have >20% long chains of DP > 20, and <20% short chains of DP < 8 (the only known exception is the semi-amylopectin of the cyanobacterium *Synechococcus* BG043511 with 25% small chains) [91]. Furthermore, with few exceptions, all plant starches have <10% short chains of DP ≤ 8 (Figure 2). It is also notable that leaf (transitory) starches have fewer longer (DP > 20) chains (approximately 20–30% of the total, compared with storage starches, which all have >40% of longer glucan chains in the granule), which presumably facilitates quick and regular turnover of starch reserves in the leaves during dark periods or stress (Figure 2).

Water-soluble phytoglycogen is a result of dysregulated branching through loss of the major debranching enzyme ISA1, which normally restricts branching frequency [76]. The resulting water-soluble particle closely resembles normal glycogen in terms of particle size and branching frequency. However, analysis of chain-length data for a range of plant phytoglycogens indicates that their chain-length distributions have a distinct clustering midway between insoluble starches and normal glycogens (Figure 2). Specifically, plant phytoglycogens show <30% of short α-glucan chains (DP ≥ 8), whereas for all normal glycogens this value is consistently above 30%. The only phytoglycogen analyzed that shows structural characteristics that are closer to normal glycogen is the isoamylase mutant (*sta7*), which makes phytoglycogen in the green alga *Chlamydomonas reinhardii* [109] (Figure 2).

### 2.2. Branching of the Glycogen Particle—Functional Implications

Unlike the water-insoluble polymers discussed in Section 2.1, glycogen particles from different kingdoms show a very wide range of branching frequency, from relatively low branching rates of 7.1% in oyster (*Ostreidae* sp.) to >18% in *Galdieria sulphuraria* [110,111,112]. Similarly, analysis of the frequencies of short (DP ≤ 8) glucan chains in glycogens from different species reveals a wide range in the proportions of short chains (from 30% to >80%; Figure 2), indicative of high degrees of structural variations between particles from different biological sources. It has been suggested that the wide variation in both branching frequency and proportions of short α-glucan chains in glycogen represents alternative survival strategies for different organisms, since these physicochemical parameters determine the size and density of particles. Particle density, specifically the distribution and frequency of shorter α-glucan chains at the particle surface, impacts degradation rates during glycogen mobilization [113,114] and energy release, which in turn underpins fitness in some organisms. Analysis of data from a number of studies with single-cell organisms (mostly prokaryotes) indicates that the average unit chain length (ACL) of glycogen correlates with survival. Specifically, cell survival time under starvation conditions increased as ACL decreased (Figure 3). Decreased ACL is associated with increased resistance to phosphorolytic degradation by GP and debranching by GDBE [113], suggesting that restricted glycogen degradation through a high density of short chains at the particle surface contributes to fitness under environmental stress, by reducing the metabolic rate. It is noteworthy that many extremophiles show very high branching frequencies (e.g., 18% α-1,6-branch linkages in *Galdieria sulphuraria*) [111,112], high proportions of short glucan chains (>60–75%), and almost no chains of ≥DP 20 (e.g., *Cyanidium caldaria* (red alga) [90] and *Sulfolobus acidocaldarius* (archaea) [115], see Figure 2). The ability of some organisms (e.g., *Anaerococcus prevotii* and *Vibrio vulnificus*) to create very short branches [116,117] (e.g., DP 3–5) means that glycogen mobilization will undoubtedly be impeded since the branched substrates at the particle surface are unsuitable substrates for GP and GDBE. Short ACL may also contribute to glycogen particle stability. Some recent studies have linked longer ACL of glycogen with fragility and instability of the particle in diabetic liver of murine models [114,118] and *E. coli* [119]. Structural analysis of glycogen particles in PGBs from Lafora disease shows that the particles have abnormal levels of long α-glucan chains, making them structurally unstable and prone to water insolubility [120].

## 3. The Role of Reversible α-Glucan Phosphorylation in Solubility and Turnover of Complex α-Polyglucans

Covalently linked phosphate monoesters (or α-glucan phosphate) are found bound to the storage α-polyglucans of many organisms. This section focuses on the mechanisms by which storage α-glucans are reversibly phosphorylated in well-studied organisms, such as plants and mammals, with some reference to red algae where information is available, and some discussion of their biological relevance. The amylopectin component of starch in certain plant tissues [132], semi-amylopectin of some Rhodophyta [92,133], and glycogen of many mammalian tissues [134] has been shown to possess glucan-bound covalently linked phosphate monoesters, the levels of which varies depending on species, tissue, or organ type. The role of α-glucan phosphate in each of these different storage carbohydrates is considered to produce localized regions of hydration within the starch granule or glycogen particle, a product of the hydrophilic negatively charged PO_4_^2−^ group, promoting access and action of hydrolytic α-glucan degrading enzymes to the surface of the starch granule in the case of water-insoluble α-glucans like starch and semi-amylopectin in order to promote turnover of the polymer.

### 3.1. α-Glucan Phosphorylation and Dephosphorylation in Plants

α-Glucan phosphorylation is quite variable in plant starches but is consistently found at highest levels in underground storage organs (tubers, rhizomes, and storage roots) of plants, comprising up to 10 nmol of G6P per mol starch [135]. While phosphate monoesters in plants are most abundant in the starch of underground storage organs (which show the B-type allomorph of structural organization) [135,136,137], they are also found, albeit in lower levels, in the transient starch of leaves and cotyledons (C-type allomorph) [138,139,140,141], and often very low or undetectable levels in storage starches of cereal endosperms (A-type allomorph) [136,138,142,143]. In plant starches, phosphate monoesters are positioned at the 6th (C6) or 3rd (C3) carbon residue of a glucosyl unit, with C6 phosphate comprising the majority (70–80%) of total phosphate monoesters found on amylopectin, and C3 comprising the remaining 20–30% [132,144]. The enzymes responsible for phosphorylation of amylopectin at the C6 and C3 sites in plants are α-glucan water dikinase (GWD; EC 2.7.9.4; also known as R1 protein) [145] and phosphoglucan water dikinase (PWD; EC 2.7.9.5), respectively, and are found as starch-granule-associated proteins [69,145].

Amylopectin phosphorylation occurs through a two-step transfer of the γ-phosphate group from ATP to a phospho-histidine domain on the C-terminus of GWD, exposing the β-phosphate group for the dikinase transfer to the C6 position of a glucosyl residue [145,146]. GWD phosphorylates at the C6 position of a glucosyl unit, which is part of a crystalline region of glucan chains, approximately eight glucosyl residues downstream of an α-1,6 glycosidic linkage [147], effectively disrupting the double helix formed by neighboring amylopectin chains [144,145,148]. The prerequisite to C3 phosphorylation by PWD is the presence of C6 phosphate on a α-glucan chain, likely priming glucan chains to be more easily accessible by PWD. Lack of GWD and C6 phosphate residues has been shown to significantly reduce the incorporation of both C6 and C3 phosphate groups on amylopectin [139], indicating that some disruption of the α-glucan helices is required for PWD function and the deposition of a C3 phosphate group [144,149]. C6 phosphate, which disrupts α-glucan helix–helix interactions, is more enriched in the amorphous regions of amylopectin, while C3 phosphate, which unwinds the helical structure of a glucan chain, is more evenly distributed across both amorphous and crystalline regions of the starch granule [135]. Studies in which GWD and PWD activity is reduced highlight the importance of α-glucan phosphate in priming the starch granule for hydrolytic degradation, as loss of GWD or PWD impairs glucan turnover and increases starch crystallinity in *gwd*^−^ mutants (caused by reduced localized hydration within the granule) [150,151,152], while the *pwd*^−^ phenotype is less severe and starch degradation is less impaired [146,153]. Unlike knockout studies, the effects of GWD overexpression are modest, despite having up to 10-fold more C6 phosphate, and this increases the proportion of long glucan chains in amorphous regions but does not greatly disrupt starch granule structure or glucan turnover [142,154].

The inability to remove α-glucan phosphate on starch has more serious implications for starch degradation and overall organism fitness. The reversible phosphorylation of plant starches plays a vital role in controlling the rate of nocturnal starch degradation by amylolytic attack, requiring the addition of covalently linked phosphate esters to push apart interacting glucan chains and increase local hydration of the surrounding area, followed by the removal of these phosphate groups to allow complete degradation of exposed α-glucan chains [155,156,157,158,159]. Glucan phosphate cleavage is achieved by two dual-specificity phosphatases (DSPs; EC 3.1.3.48), Starch EXcess 4 (SEX4) and Like Starch Excess Four 2 (LSF2), which cleave C6 and C3 phosphate, or C3 phosphate only, respectively. Loss of either of these DSPs results in an excess starch phenotype in Arabidopsis leaves, an inability to degrade transient starch during the dark period [140,151,160]. Removal of these phosphate groups allows for complete metabolism of an α-glucan chain by the combined actions of β-amylase (BAM; EC 3.2.1.2) and isoamylase. The presence of α-glucan phosphate also prevents the degradation of amylopectin chains by BAM beyond a phosphorylated glucosyl residue, and hence these phosphate groups need to be cleaved for further starch turnover [161]. SEX4 possesses a carbohydrate-binding module-48 (CBM48) domain to facilitate substrate binding, and a DSP domain for phosphatase activity at both the C6 and C3 glucosyl sites [162], while LSF2 does not have a CBM module but utilizes secondary binding sites to interact with its substrate [163] to remove C3 phosphate residues [140] (Figure 4). A LSF2 homolog, termed *Ostta*DSP, was identified in the primitive micro-alga *Ostreococcus tauri*, indicative of α-glucan phosphorylation as a means of regulating diurnal starch turnover in ancestral lineages [164]. LSF1 is a non-catalytic member of the α-glucan phosphatase family and acts as a scaffold by recruiting BAMs to the granule surface for amylolytic attack [165]. While the role of phosphate monoesters in the process of starch biosynthesis is less clear, amylopectin chains are phosphorylated during starch synthesis [166,167,168,169], and C6 phosphate residues in particular appear to be important for establishing the amorphous regions of the starch granule [142,154].

Studies in potato (*Solanum tuberosum* L.) tuber starches suggest phosphate monoesters accumulate on longer chains, likely those in the amorphous regions of the granule [176,177]. The variation in the levels of phosphate monoesters in different plant tissues ultimately is reflected in the differences in the crystalline allomorphs observed, i.e., cereal grain starch, a form of long-term storage, which have few phosphate monoesters and a distinctly different crystallinity pattern (A-allomorph) than that of highly phosphorylated starch in underground storage organs (B-allomorh), and is also different from intermediately phosphorylated transient starch (C-allomorph) [82]. In general, there is a strong correlation between phosphate monoester levels in starches and the unit chain length of amylopectin; typically, starches with long chain lengths and low branching frequency show high levels of α-glucan-bound phosphate [136,178]. Interestingly, if branching activity is suppressed, e.g., in potato tuber, and leaves of Arabidopsis (*Arabidopsis thaliana* L.) and canola (*Brassica napus* subsp. *napus*), there is a corresponding increase in phosphate monoesters in the starch [141,179,180], illustrating a negative correlation between α-glucan branching and phosphate monoester content. The presence of monophosphate esters has been shown to reduce the substrate affinities of branching enzymes for α-glucan substrates [181]. Some of the observed variability in starch phosphate levels of plants is thought to be related to species-specific differential catalytic activities of SEX4. For example, cereal endosperm tissue (low-starch phosphate) shows significantly higher measurable SEX4 activity than potato tuber or rhizome tissue (high-starch phosphate) [182].

### 3.2. α-Glucan Phosphorylation and Dephosphorylation in Rhodophytes

Within the Rhodophyta, storable carbohydrate energy exists as semi-amylopectin [92,93], semi-amylopectin and amylose in the Porphyridiales [90], or glycogen [90,93,94,111], though semi-amylopectin biosynthesis appears to be the primary form of bulk carbohydrate storage for most organisms in this phylum (see Section 1.2). Red algae signify the interesting divergence of two predominant types of storage carbohydrates (starch and glycogen) across eukaryotic organisms. Semi-amylopectin structure and turnover in red algae is relatively understudied, but the presence of homologous α-glucan phosphorylation machinery to that of plants and green algae (Viridaplantae) suggests that semi-amylopectin α-glucan phosphate most likely contributes a similar role for glucan turnover as that of plant starch. Due to the lack of sequenced nuclear genomes in red algae, it is largely unknown whether all red algae that store semi-amylopectin show reversible glucan phosphorylation. Some species which make glycogen (class Cyanidiophyceae) lack the biosynthetic machinery required for reversible α-glucan phosphorylation [93,94], while one Cyanidiophore (*Cyanidioschyzon merolae*) instead makes phosphorylated semi-amylopectin. The presence of α-glucan phosphate on semi-amylopectin has been demonstrated in only two red algal species, *C. merolae* (unicellular extremophile) and *Gracilariopsis lemaneiformis* (red-brown coastal seaweed) [92,133]. In *C. merolae*, 95% of phosphate monoesters are found at the C6 position, due to the presence of only a GWD homolog, which is responsible for C6 phosphorylation, and not PWD, required for C3 phosphorylation [133]. Loss of GWD in *C. merolae* effectively prevents the accumulation of C6 phosphate residues on semi-amylopectin, ultimately slowing the rate at which this cytosolic polyglucan can be degraded, with cell survival compromised under prolonged darkness and nutrient deprivation conditions compared to the wild-type (WT) [133]. Interestingly, recent evidence shows that GWD-mediated starch turnover in *C. merolae* is regulated by protein phosphorylation involving the target of rapamycin (TOR) signaling pathway [183]. The proportions of C6 to C3 phosphate remain unknown for species that possess both GWD and PWD homologs, such as *G. lemaneiformis* [92]. Other species of red algae (e.g., *Neoporphyra haitensis*, *Chondrus crispus*, *Gracilariopsis chorda*, and *Porphyridium purpureum*) also have identified putative PWD homologs [184], suggesting that these species may also possess semi-amylopectin-bound C3 phosphate (Figure 4). However, the contributions of C3 phosphate to glucan turnover in red algae are unknown, and alterations in the proportions of C6 and C3 phosphate may ultimately affect the extent to which semi-amylopectin can be hydrated.

Sufficient data are lacking on the phosphate monoesters of red algae storage polyglucans to draw significant conclusions on the levels of α-glucan-bound phosphate found across this vast phylum, and whether the variation in α-glucan phosphate (and likely other structural features of semi-amylopectin) may play a role in determining organism fitness overall and dictate the particular environment in which a species may proliferate. However, semi-amylopectin crystallinity is the same as that of transient starch (C-type allomorph) [92] and phosphate monoester levels are also comparable to leaf and cotyledon starch, and thus likely share a similar mechanism for diurnal α-glucan turnover as transient starch. While semi-amylopectin phosphorylation remains relatively understudied in red algae, similar to plants, phosphate monoesters in red algae have been shown to play an important role in α-glucan turnover in the dark and in times of stress [133,183,185]. Glucan dephosphorylation of semi-amylopectin is achieved by a functional equivalent of SEX4, laforin, which cleaves both C6 and C3 phosphate groups [186,187,188]. Red algae laforin is structurally more homologous with the laforin of animalia, and similarly forms oligomers, unlike SEX4 from plants which is monomeric [188]. While reversible phosphorylation remains relatively understudied in red algae, at least seven species have been identified to possess laforin homologs [184], all containing the canonical DSP domain, but with 1–3 repetitive CBM20 domains (Figure 4). The reason for variation in the CBM20 domain number remains unclear, including whether this variation plays a role in determining the rate at which each homolog dephosphorylates semi-amylopectin; however, based on in silico analyses, some of these additional CBM20 domains are thought to be non-functional and the result of CBM20 duplication [184,189]. Unlike most of the studied Rhodophyte species, *Galdieria sulphuraria* and *Cyanidium caldarium* (class Cyanidiophyceae) synthesize glycogen [93,94], and possess neither GWD, PWD, nor laforin homologs.

Several protists (apicomplexans and cilliophores), which store glycogen, and a cryptomonad (*Guillardia theta*), which stores starch, also have identified putative laforin homologs [187,189], which suggests that the α-polyglucans of these organisms possess phosphate monoesters, though the fine structure of glycogen particles or starch granules made, including degree of α-glucan phosphorylation, is largely unknown. Similarly, putative GWD, PWD, and laforin homologs have been identified in the genome of the glaucophyte *Cyanophora paradoxa*, but little is known about the contribution of these enzymes toward starch structure and turnover in the cytosol [156,184].

### 3.3. α-Glucan Phosphorylation and Dephosphorylation in Animals

The role of α-glucan phosphate addition and turnover in glycogen of animals is much less clear. Glucan-bound phosphate is present in substantial quantities in the glycogen of muscle and organ tissues at C2, C3, and C6 glucosyl residues [134,190,191]. Glycogen phosphate plays a role in maintaining particle solubility by disrupting the α-glucan helical structure, preventing glucan–glucan chain association. While C6 phosphorylation is the most abundant phosphate ester in starch and semi-amylopectin, it constitutes a relatively minor portion (~20%) of α-glucan-bound phosphate in glycogen compared to C3 and C2 phosphate esters (~40% each) [192] (Figure 4). C6 phosphate is found to be more abundant near the center of the glycogen particle and on longer chains, and thus likely plays a role in maintaining structural integrity and solubility of the inner particle [190,193]. α-Glucan phosphorylation may, therefore, play a complementary role with branching by maintaining hydration of the glycogen polymer.

The proportion of glucan-bound phosphate found in glycogen varies across tissue types (i.e., brain, liver, and muscle) and is found to be highly phosphorylated in muscle and brain tissue. Variation in levels of α-glucan phosphorylation observed in different tissues and organ types may point to specific aspects of metabolic regulation of glycogen stores, as seen with variations in starch phosphate in different plant tissues (Section 3.1 above). The laforin phosphatase regulates glycogen phosphate levels and is in turn regulated by AMP-activated protein kinase [194], suggestive of a link between the regulation of α-glucan phosphate turnover and cellular energy status. It has been suggested that α-glucan phosphate addition, particularly in tissues with high glycogen phosphate content, acts as a regulatory control on the degradative enzymes GP and GDBE [195,196]. By limiting complete degradation of α-1,4-chains and α-1,6-branch linkages, the presence of phosphate esters may ensure that the central core of the particle remains to facilitate rapid resynthesis of the glycogen particle, which may be advantageous in tissues such as skeletal muscle following strenuous exercise [3,195]. Interestingly, it has been shown that phosphorylated glycogen is a more effective substrate for GS, consistent with the observed rapid synthesis of glycogen particles following prolonged, exhaustive exercise [195,197].

The enzyme(s) or mechanism by which phosphate monoesters are incorporated into glycogen is widely considered to be elusive, with no known functional equivalent of GWD or PWD present in animals. Lomako et al. (1993) [198] described a UDP-Glc:glycogen Glc 1-phosphotransferase activity in animal cells, which they posited as being responsible for glycogen phosphorylation, although no subsequent studies have been published in support of this. However, despite lack of progress identifying the agents responsible for glycogen phosphorylation, the removal of C2, C3, and C6 phosphate esters by the DSP laforin is well studied. Loss of laforin, or interaction proteins such as malin (an E3 ubiquitin ligase of laforin) and PR/SET domain 8 (PRDM8, a nuclear translocator of laforin and malin), leads to Lafora’s disease [199,200,201]. Lafora’s disease is the accumulation of water-insoluble polyglucosan bodies (PGBs, or Lafora bodies) that have a higher proportion of long glucan chains than WT glycogen [120,202,203,204,205] in addition to elevated levels of phosphate monoesters [192,206]. Lafora bodies are more resistant to enzymatic degradation due to the inability to remove α-glucan-bound phosphate, which prevents proper storage and degradation of glycogen and results in progressive myoclonus epilepsy and eventual death in young adults [207,208]. While the absence of laforin in mice causes the formation of insoluble PGBs, the overexpression of a catalytically inactive laforin in mice results in the synthesis of a water-soluble yet hyperphosphorylated form of glycogen, indicating that the stability of the laforin–malin interaction is critical for maintaining polymer solubility [205,209,210]. The critical factor in glycogen insolubility was determined to be not the hyperphosphorylation of glycogen per se, but the long chains that are formed on the particle, which ultimately leads to water insolubility, presumably through α-glucan chain interactions, this being a causal agent of the inability for α-glucan phosphate to be removed [205]. Under normal conditions, it has been proposed that glycogen phosphate addition acts as a signal for quality control, since if glycogen phosphate accumulates, the particle becomes less structurally stable, more water-insoluble, and prone to degradation via the lysosome [196,211]. Animal laforin and plant SEX4 share close structural and functional relationships, to the extent that human (*Homo sapiens*) laforin can complement plant *sex4*^−^ mutants by dephosphorylating plant starch and restoring normal growth [186].

While mammalian glycogen is well studied, there appears to be a lack of laforin in certain animal lineages, such as insects and worms, as well as in fungi and prokaryotes [187], which might suggest that glycogen in these organisms lacks phosphate esters. Interestingly, a study by Viksø-Nielsen et al. [212] showed that expression of the plant R1 enzyme (GWD) in *E. coli* cells caused a five-fold increase in glycogen phosphate compared with low levels of phosphate monoesters (0.9 nmol/mg glycogen) detected in wild-type *E. coli* glycogen. Further studies are needed to determine whether glycogen phosphorylation in prokaryotic cells is a general phenomenon, and how this process is mediated in the absence of known glucan phosphorylation machinery.

## 4. Summary and Conclusions

The distinct physicochemical properties of starch, starch-like polymers, and glycogen arise primarily from the control of α-1,6-branch points and their distribution to facilitate the formation of, respectively, water-insoluble or water-soluble polymers. Starch granules are a high caloric density long-term carbon store formed through restricted branching and debranching to produce an architecturally complex structure resistant to hydrolysis. Access of hydrolytic enzymes (BAM and AMY) for starch degradation requires loosening of tight water-resistant glucan–glucan interactions by the insertion of charged, hydrophilic phosphate groups by GWD and PWD. Efficient turnover of starch and starch-like polymers also requires phosphate monoester removal by DSPs. The open, water-soluble glycogen particle is characterized by high branching frequency and shorter α-glucan chains. In simple unicellular organisms, restricting access of these carbon reserves by the phosphorolytic action of GP may be via excessive branching at the polymer surface. Phosphate groups are found in glycogen at lower frequencies than starch, and the agents responsible for glycogen phosphorylation are unknown. Although the precise biological role of glycogen phosphorylation is unclear, its presence may induce particle stability by maintaining hydration, particularly for the inner regions of the particle, thereby preventing water insolubility. It is not known whether glycogen phosphorylation is a general phenomenon across all kingdoms.

## Figures and Tables

**Figure 1:**
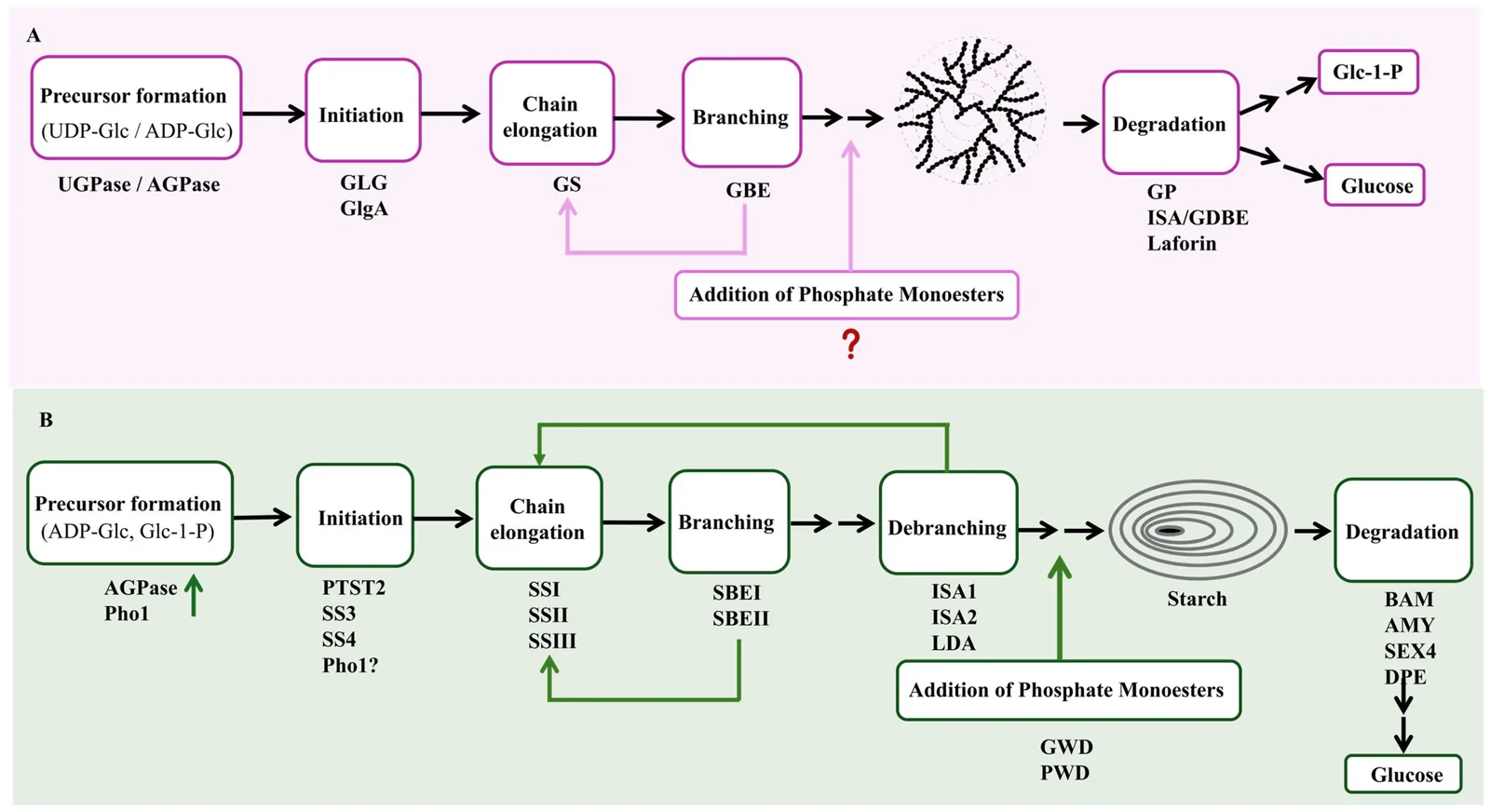
Simplified comparative schematic pathways of glycogen metabolism in eukaryotic and prokaryotic cells (**A**), and starch synthesis and degradation in plant and green algal cells (**B**). Abbreviations used: ADP-Glc, ADP-glucose; AGPase, ADP-Glc pyrophosphorylase; AMY, α-amylase(s); BAM, β-amylase(s); DPE, disproportionating enzyme; GBE, glycogen branching enzyme; GDBE, glycogen debranching enzyme; Glc1P, glucose 1-phosphate; GLG, glycogenin; GlgA, microbial glycogen synthase; GP, glycogen phosphorylase; GS, glycogen synthase; GWD, α-glucan water dikinase; ISA, isoamylase; LDA, limit dextrinase; Pho1, plastidial starch phosphorylase; PTST2, protein targeting to starch 2; PWD, phosphoglucan water dikinase; SBE, starch branching enzyme; SS, starch synthase; UDP-Glc, UDP-glucose; UGPase, UDP-Glc pyrophosphorylase. The mechanism responsible for the phosphorylation of glycogen (**A**) is unknown and, therefore, denoted by a question mark.

**Figure 2:**
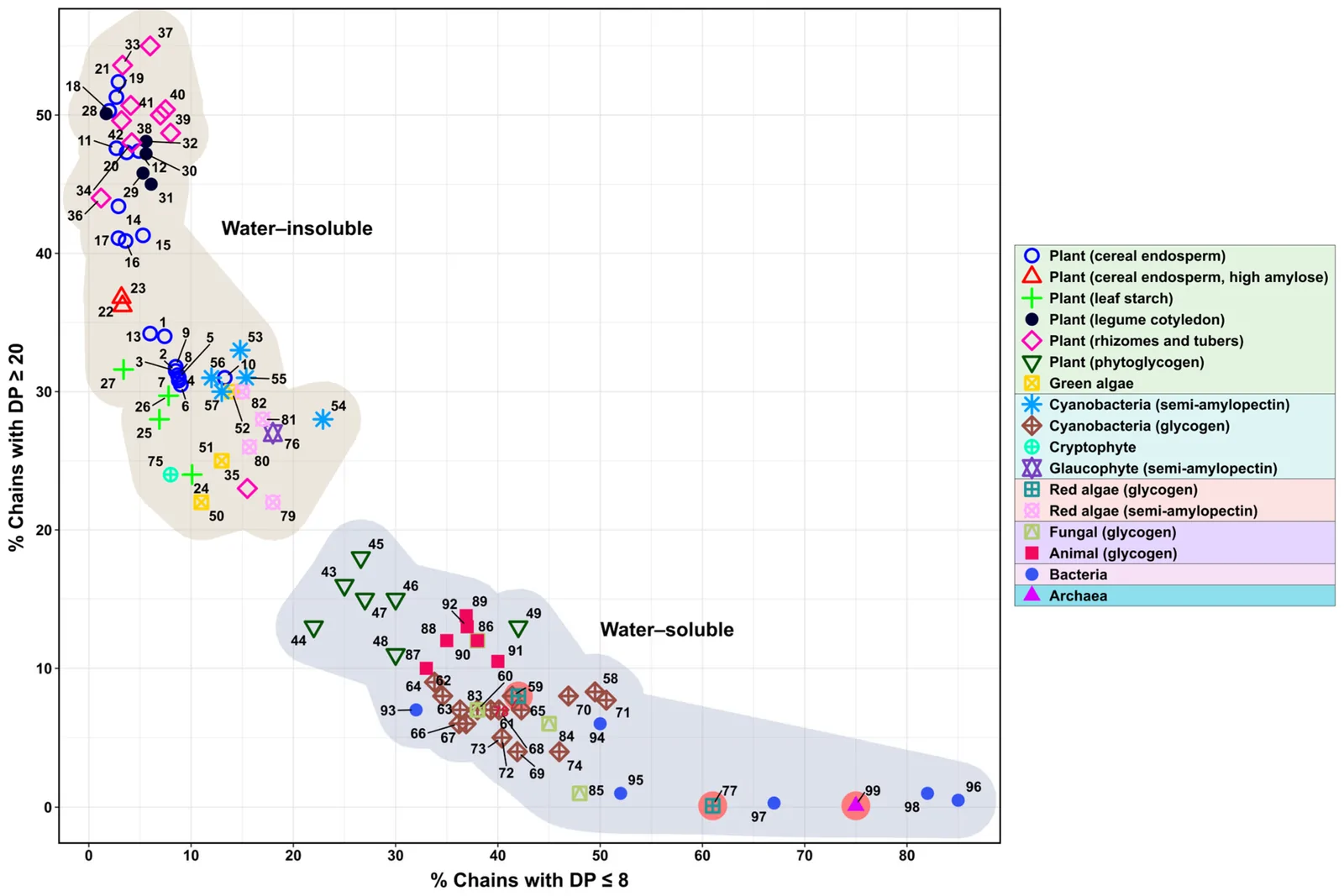
Structural characteristics of various natural α-polyglucans, which determine their cellular solubility. Plot shows chain-length distribution data of starches, glycogens, phytoglycogens, and floridean starches/semi-amylopectins from various biological sources, and the relationship between the proportion of long α-1,4-glucan chains (≥20 DP) and that of short α-1,4-glucan chains (≤8 DP) found in each polymer in relation to their degree of water solubility. Water-insoluble starches of plants and green algae, semi-amylopectins (cyanobacteria, glaucophytes and select Rhodophytes), and floridean starches of Rhodophytes (red algae) all have >20% chains of longer than 20 DP. The color-coded key on the right of the figure shows various symbols used in the plot grouped according to major classes of organisms and their respective major storage α-polyglucan in parentheses: plants and green algae (green), cyanobacteria, cryptophytes, and glaucophytes (pale blue), Rhodophytes (red), animals and fungi (purple), bacteria (pink), and archaea (dark blue). Symbols in orange circles (#59, #77, and #99) refer to extremophile organisms. Individual numerical data points refer to studies referenced in Supplementary Table S1.

**Figure 3:**
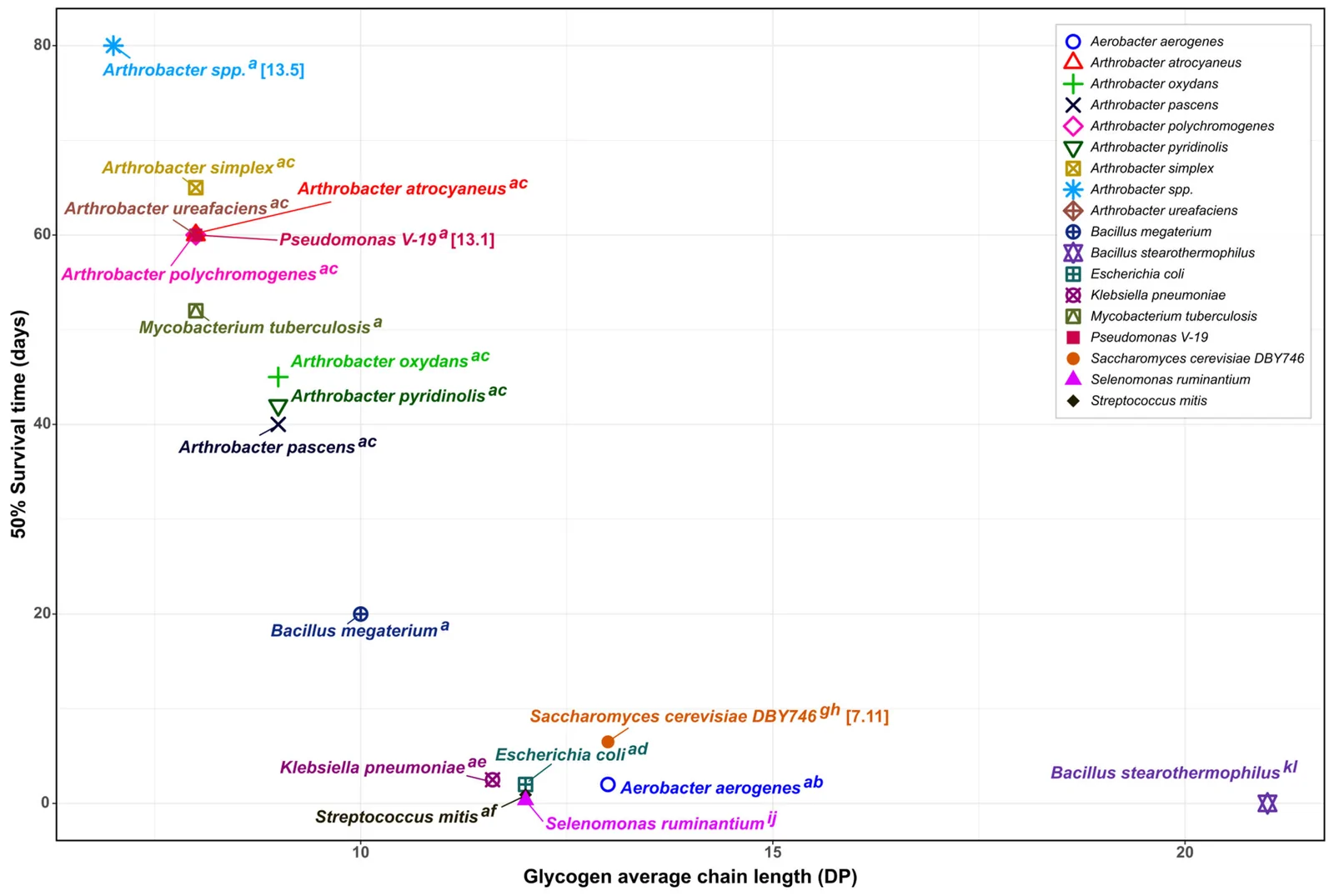
Relationship between fitness of some single-cell organisms and glycogen structure, determined as survival time plotted against the average chain length (ACL, measured as DP) of glycogen reserves. Each data point on the plot refers to a single study of a specific organism, as indicated, and the accompanying italicized superscript letter refers to the corresponding study: *^a^* [113], *^b^* [121], *^c^* [122], *^d^* [123], *^e^* [124], *^f^* [125], *^g^* [126], *^h^* [127], *^i^* [128], *^j^* [129], *^k^* [130], and *^l^* [131]. Percentage values in parentheses are determinations of branching frequency of glycogen for each respective organism, where known.

**Figure 4:**
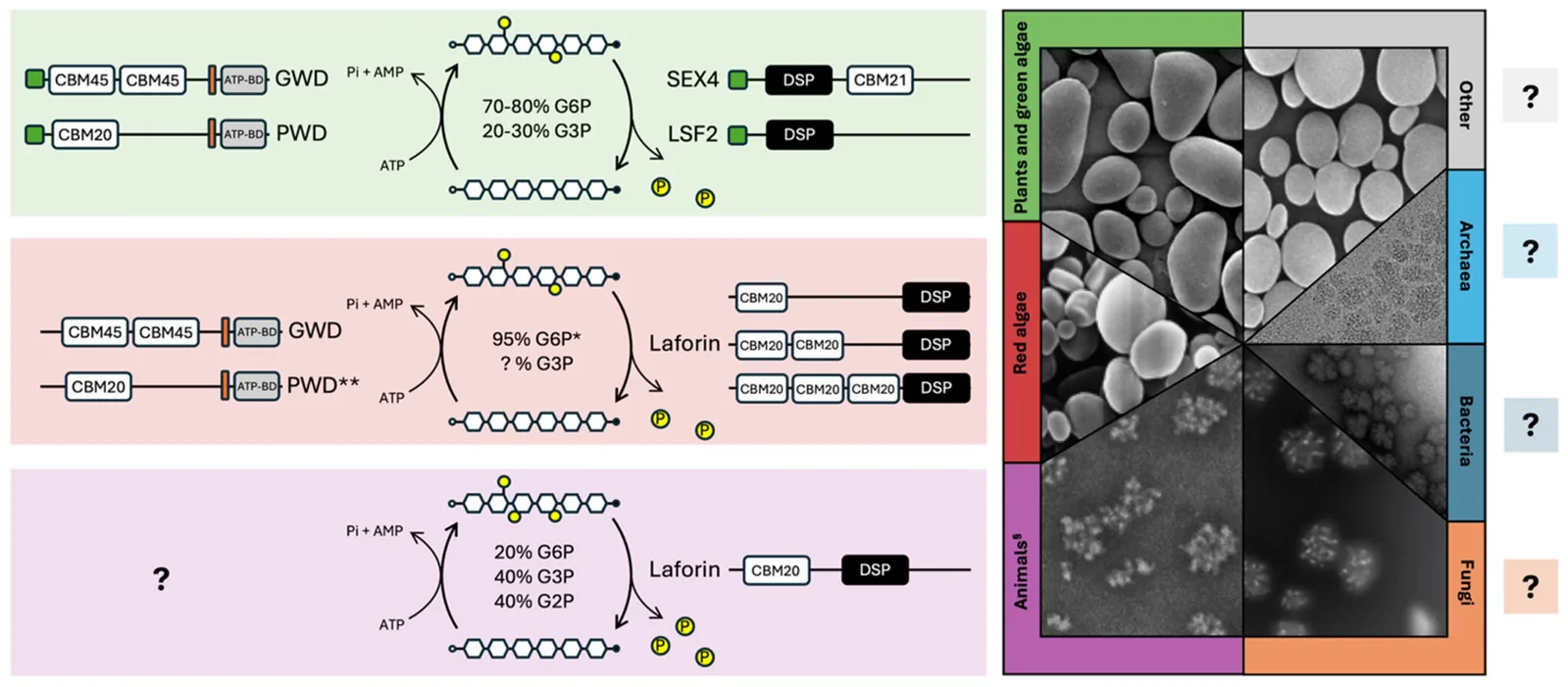
Schematic depicting the known mechanisms by which storage α-polyglucans are reversibly phosphorylated. In plants and green algae, amylopectin chains are phosphorylated by the α-glucan water dikinases (GWD and PWD), which bind to starch via a CBM domain. ATP binds the ATP-binding domain (orange box) and donates the β-phosphate group to a glucosyl residue. Phosphate esters are covalently linked to the C6 or C3 carbon of a glucosyl unit, by GWD and PWD, respectively. Phosphate esters are cleaved by dual-specificity phosphatases (DSPs) SEX4 and LSF2: SEX4 cleaves C6 and C3 phosphate esters, with LSF2 cleaving only C3 phosphate esters. These enzymes are plastid-localized (green box indicates N-terminal plastid targeting transit peptide). In red algae, semi-amylopectin made in the cytosol has been shown to be phosphorylated at the C6 position by GWD in *Cyanidioschyzon merolae*. ** Annotations of PWD have been made in some red algae such as *Neoporphyra haitanensis*, but have not been validated, nor has C3 phosphate been quantified in semi-amylopectin. Red algae have the functional equivalent of SEX4, the DSP laforin, which can have up to three CBMs. In animals (* not including insects), glycogen is phosphorylated at the C6, C3, and C2 sites, but the mechanism by which glycogen phosphorylation occurs is unknown (denoted by question mark). Laforin is known to dephosphorylate glycogen at all three positions (C2, C3, and C6). It is unknown whether other glycogen synthesizing organisms (fungi, bacteria, and archaea) and other floridean starch synthesizing organisms (such as glaucophytes and dinoflagellates) possess glucan-bound phosphate, and these are denoted by a question mark inside a box of the corresponding color in the diagram. Lack of laforin or SEX4 homologs in these organisms (except for in the glaucophyte *Cyanophora paradoxa*) suggests storage carbohydrates from these organisms are likely not phosphorylated. Other organisms, such as cilliophores and cryptomonads, have identified laforin homologs, but the mechanism of α-glucan phosphorylation for these organisms remains unidentified. Structural enzyme diagrams are not drawn to scale. Sources of electron microscope images in the figure are as follows: plant and green algae, potato starch [170]; other, dinoflagellate floridean starch (*Crypthecodinium cohnii*) [171]; archaea, *Sulfolobus* glycogen [172]; bacteria, *E. coli* glycogen [173]; fungi, Shiitake mushroom (*Lentinula edodes*) glycogen [174]; animal, mouse liver glycogen [175]; red algae, *Gracilariopsis* sp. semi-amylopectin [92].

## Data Availability

No new data were created or analyzed in this study. Data sharing is not applicable to this article.

## Supplementary Materials

The following supporting information can be downloaded at: https://www.mdpi.com/article/10.3390/molecules31193453/s1, Table S1: Chain-length (CL) distribution of α-polyglucans from plants, algae, cyanobacteria, bacteria, archaea, and animals. References [31,80,88,90,91,92,94,103,109,111,115,213,214,215,216,217,218,219,220,221,222,223,224,225,226,227,228] are cited in the Supplementary Materials.
