# Supplementary material for: Solving the Insoluble: Regulation of α-Polyglucan Solubility and Functionality in Nature
Source: Molecules. 2026 Sep 28;31(19):3453. doi: 10.3390/molecules31193453 (PMC13666283; doi:10.3390/molecules31193453)
Supplement: Supplementary file 1 [file molecules-31-03453-s001.zip › molecules-4564841-supplementary.pdf]

Supplementary Table S1: Chain-length (CL) distribution of α-polyglucans from plants, algae, cyanobacteria, bacteria, archaea, and animals.

*Refers to different rice genotypes.

Category	Sample	Solubility	Sample number	ΣDP ≤ 8 (mole %)	ΣDP ≥ 20 (mole %)	Ref.
Plant (cereal endosperm, starch)	Rice (*Oryza sativa* L.) cv. Kinmaze	Insoluble	#1	7.42	34	[91]
Plant (cereal endosperm, amylopectin)	*Waxy Kanto 107*4/K107Wx1-nil	Insoluble	#2	8.5	31.5	[213]
Plant (cereal endosperm, amylopectin)	*Waxy Kanto 107*4/K107Wx2-nil	Insoluble	#3	8.5	31.5	[213]
Plant (cereal endosperm, starch)	*Non-waxy K107Wx1-nil	Insoluble	#4	8.8	31	[213]
Plant (cereal endosperm, starch)	*Non-waxy K107Wx2-nil	Insoluble	#5	8.8	31	[213]
Plant (cereal endosperm, starch)	*K107Afpp1	Insoluble	#6	9	30.5	[213]
Plant (cereal endosperm, starch)	*Tanikei A6099	Insoluble	#7	8.8	30.8	[213]
Plant (cereal endosperm, starch)	*K107Afpp4	Insoluble	#8	8.7	31.2	[213]
Plant (cereal endosperm, starch)	*Tanikei A6599-4	Insoluble	#9	8.5	31.8	[213]
Plant (cereal endosperm, starch)	Maize (*Zea mays* L.)	Insoluble	#10	13.3	31	[31]
Plant (cereal endosperm, starch)	Normal maize	Insoluble	#11	2.7	47.6	[103]
Plant (cereal endosperm, amylopectin)	Waxy maize	Insoluble	#12	4.9	47.4	[103]
Plant (cereal endosperm, amylopectin)	Waxy rice	Insoluble	#13	6	34.2	[103]
Plant (cereal endosperm, starch)	Normal rice	Insoluble	#14	2.9	43.4	[103]
Plant (cereal endosperm, starch)	Sweet rice	Insoluble	#15	5.3	41.3	[103]
Plant (cereal endosperm, starch)	Wheat (*Triticum aestivum* L.)	Insoluble	#16	3.6	40.9	[103]
Plant (cereal endosperm, starch)	Cattail millet (*Pennisetum glaucum* L.)	Insoluble	#17	2.9	41.1	[103]
Plant (cereal endosperm, starch)	Normal corn (maize)	Insoluble	#18	2	50.3	[214]
Plant (cereal endosperm, amylopectin)	Waxy corn (maize)	Insoluble	#19	2.7	51.3	[214]
Plant (cereal endosperm, starch)	W.B. Merlin barley (*Hordeum vulgare* L.)	Insoluble	#20	3.7	47.3	[215]
Plant (cereal endosperm, starch)	Glacier barley	Insoluble	#21	2.9	52.4	[215]
Plant (cereal endosperm, high amylose, starch)	Ingredion Hylon V starch (maize)	Insoluble	#22	3.3	36.2	[216]
Plant (cereal endosperm, high amylose, starch)	Ingredion Hylon VII starch (maize)	Insoluble	#23	3.2	36.8	[216]
Plant (leaf, starch)	Arabidopsis (*Arabidopsis thaliana* L.)	Insoluble	#24	10.1	24	[217]
Plant (leaf, starch)	Wheat leaf starch	Insoluble	#25	6.9	28	[216]
Plant (leaf, starch)	Maize leaf starch	Insoluble	#26	7.8	29.7	[216]
Plant (leaf, starch)	Barley leaf starch	Insoluble	#27	3.4	31.6	[216]
Plant (legume cotyledon, starch)	Mung bean (*Vigna radiata* L.)	Insoluble	#28	1.7	50.1	[103]

Plant (legume cotyledon, starch)	Carneval pea (*Pisum sativum* L.)	Insoluble	#29	5.3	45.8	[218]
Plant (legume cotyledon, starch)	Carrera pea	Insoluble	#30	5.6	47.2	[218]
Plant (legume cotyledon, starch)	Grande pea	Insoluble	#31	6.1	45	[218]
Plant (legume cotyledon, starch)	Keoma pea	Insoluble	#32	5.6	48.1	[218]
Plant (rhizomes and tubers, starch)	Tapioca (*Manihot esculenta* Crantz)	Insoluble	#33	3.3	53.6	[103]
Plant (rhizomes and tubers, starch)	Chinese taro (*Colocasia esculenta* (L.) Schott)	Insoluble	#34	4.2	48	[103]
Plant (rhizomes and tubers, starch)	Potato (*Solanum tuberosum* L.) amylopectin	Insoluble	#35	15.5	23	[31]
Plant (rhizomes and tubers, starch)	Turmeric rhizome (*Curcuma longa* L.)	Insoluble	#36	1.2	44	[216]
Plant (rhizomes and tubers, starch)	Normal potato	Insoluble	#37	6	55	[219]
Plant (rhizomes and tubers, starch)	Waxy potato	Insoluble	#38	7	50	[219]
Plant (rhizomes and tubers, starch)	Yam (*Dioscorea* sp.)	Insoluble	#39	8	48.7	[219]
Plant (rhizomes and tubers, starch)	Sweet potato (*Ipomoea batatas* (L.) Lam.)	Insoluble	#40	7.5	50.4	[219]
Plant (rhizomes and tubers, starch)	Water chestnut (*Eleocharis dulcis)*	Insoluble	#41	4.1	50.7	[103]
Plant (rhizomes and tubers, starch)	Lotus root (*Nelumbo nucifera*)	Insoluble	#42	3.2	49.6	[103]
Plant (phytoglycogen)	Sweet maize, *su1*	Soluble	#43	25	16	[88]
Plant (phytoglycogen)	*hvisa1-3* barley mutant	Soluble	#44	22	13	[220]
Plant (phytoglycogen)	Rice *sugary-1* mutant EM914	Soluble	#45	26.6	18	[91]
Plant (phytoglycogen)	Arabidopsis *Atisa1-1* (N542704)	Soluble	#46	30	15	[80]
Plant (phytoglycogen)	Arabidopsis *Atisa2-1* (CTI15)	Soluble	#47	27	15	[80]
Plant (phytoglycogen)	Arabidopsis *Atisa2-1/Atpu1-1* (5FWB6)	Soluble	#48	30	11	[80]
Plant (phytoglycogen)	*Chlamydomonas reinhardtii sta7* mutant	Soluble	#49	42	13	[109]
Green algae (starch)	*Chlamydomonas reinhardtii*	Insoluble	#50	11	22	[221]
Green algae (starch)	*Ostreococcus tauri*	Insoluble	#51	13	25	[222]
Green algae (starch)	*Chlorella sorokiniana*	Insoluble	#52	14	30	[223]
Cyanobacteria (semi-amylopectin)	*Cyanobacterium* sp. MBIC10216	Insoluble	#53	14.8	33	[91]
Cyanobacteria (semi-amylopectin)	*Myxosarcina burmensis*	Insoluble	#54	22.9	28	[91]
Cyanobacteria (semi-amylopectin)	*Synechococcus* sp. BG043511	Insoluble	#55	15.4	31	[91]
Cyanobacteria (semi-amylopectin)	*Cyanothece* sp. ATCC 51142	Insoluble	#56	12	31	[224]
Cyanobacteria (semi-amylopectin)	*Cyanobacterium* sp. CLg1	Insoluble	#57	13	30	[224]
Cyanobacteria (glycogen)	*Anabaena* sp. PCC7120	Soluble	#58	49.5	8.3	[91]
Cyanobacteria (glycogen)	*Synechococcus* sp. MBIC10456	Soluble	#59	41.4	8	[91]
Cyanobacteria (glycogen)	*Synechococcus* sp. MBIC10459	Soluble	#60	38	7	[91]
Cyanobacteria (glycogen)	*Dermocarpa* sp. MBIC10001	Soluble	#61	39.3	7	[91]
Cyanobacteria (glycogen)	*Dermocarpa* sp. MBIC10766	Soluble	#62	34.6	8	[91]

Cyanobacteria (glycogen)	*Dermocarpa* sp. MBIC10768	Soluble	#63	36.3	7	[91]
Cyanobacteria (glycogen)	*Fischerella major* NIES-N-592	Soluble	#64	33.8	9	[91]
Cyanobacteria (glycogen)	*Spirulina platensis* IAM-M-135	Soluble	#65	42.3	7	[91]
Cyanobacteria (glycogen)	*Synechococcus* MBIC10073	Soluble	#66	36.2	6	[91]
Cyanobacteria (glycogen)	*Synechococcus* sp. MBIC10079	Soluble	#67	36.9	6	[91]
Cyanobacteria (glycogen)	*Synechococcus* sp. LPP-group MBIC10597	Soluble	#68	40.1	7	[91]
Cyanobacteria (glycogen)	*Synechococcus* MBIC10089	Soluble	#69	41.9	4	[91]
Cyanobacteria (glycogen)	*Oscillatoria limnetica* NIES-N-36	Soluble	#70	46.9	8	[91]
Cyanobacteria (glycogen)	*Phormidium* sp. MBIC10003	Soluble	#71	50.6	7.7	[91]
Cyanobacteria (glycogen)	*Phormidium* sp. MBIC10070	Soluble	#72	40.4	5	[91]
Cyanobacteria (glycogen)	*Phormidium* sp. MBIC10076	Soluble	#73	40.4	5	[91]
Cyanobacteria (glycogen)	*Synechococcus* sp. PCC7942	Soluble	#74	46	4	[90]
Cryptophyte (starch)	*Guillardia theta*	Insoluble	#75	8	24	[221]
Glaucophyte (semi-amylopectin)	*Cyanophora paradoxa*	Insoluble	#76	18	27	[225]
Red algae (glycogen)	*Galdieria sulphuraria*	Soluble	#77	61	0.1	[94]
Red algae (glycogen)	*Cyanidium caldarium*	Soluble	#78	42	8	[90]
Red algae (semi-amylopectin)	*Porphyridium purpureum*	Insoluble	#79	18	22	[90]
Red algae (semi-amylopectin)	*Rhodosorus marinus*	Insoluble	#80	15.7	26	[94]
Red algae (semi-amylopectin)	*Rhodella violacea*	Insoluble	#81	17	28	[94]
Red algae (semi-amylopectin)	*Gracilariopsis lemaneiformis*	Insoluble	#82	15	30	[92]
Fungi (glycogen)	*Volvariella volvacea*	Soluble	#83	38	7	[226]
Fungi (glycogen)	*Aspergillus fumigatus*	Soluble	#84	45	6	[226]
Fungi (glycogen)	*Saccharomyces cerevisiae*	Soluble	#85	48	1	[216]
Fungi (glycogen)	*Ophiocordyceps sinensis*	Soluble	#86	38	12	[227]
Animal (glycogen)	Slipper limpet (*Crepidula fornicata*)	Soluble	#87	33	10	[226]
Animal (glycogen)	Mussel (*Mytilus edulis*) glycogen	Soluble	#88	35	12	[226]
Animal (glycogen)	Bovine (*Bos taurus*) liver	Soluble	#89	36.9	13.8	[228]
Animal (glycogen)	Rabbit (*Oryctolagus cuniculus*) liver	Soluble	#90	38	12	[228]
Animal (glycogen)	Bovine liver	Soluble	#91	40	10.5	[227]
Animal (glycogen)	Oyster (*Crassostrea gigas*)	Soluble	#92	37	13	[228]
Bacteria (glycogen)	*Escherichia coli*	Soluble	#93	32	7	[111]
Bacteria (glycogen)	*Sphaerotilus natans*	Soluble	#94	50	6	[111]
Bacteria (glycogen)	*Mycobacterium bovis* BCG	Soluble	#95	52	1	[226]
Bacteria (glycogen)	*Arthrobacter viscous*	Soluble	#96	85	0.5	[111]
Bacteria (glycogen)	*Pseudomonas* V-19	Soluble	#97	67	0.3	[111]

Bacteria (glycogen)	*Arthrobacter globiformis*	Soluble	#98	82	1	[111]
Archaea (glycogen)	*Sulfolobus acidocaldarius*	Soluble	#99	75	0.1	[115]
